# Overexpression of D-Xylose Reductase (*xyl*1) Gene and Antisense Inhibition of D-Xylulokinase (*xyiH*) Gene Increase Xylitol Production in *Trichoderma reesei*

**DOI:** 10.1155/2014/169705

**Published:** 2014-06-11

**Authors:** Yuanyuan Hong, Mehdi Dashtban, Greg Kepka, Sanfeng Chen, Wensheng Qin

**Affiliations:** ^1^Department of Biology, Lakehead University, Thunder Bay, ON, Canada P7B 5E1; ^2^State Key Laboratory for Agrobiotechnology and College of Biological Sciences, China Agricultural University, Beijing 100193, China; ^3^School of Environmental Sciences, University of Guelph, Guelph, ON, Canada N1G 2W1; ^4^Instrumentation Laboratory, Lakehead University, 955 Oliver Road, Thunder Bay, ON, Canada P7B 5E1

## Abstract

*T. reesei* is an efficient cellulase producer and biomass degrader. To improve xylitol production in *Trichoderma reesei* strains by genetic engineering, two approaches were used in this study. First, the presumptive D-xylulokinase gene in * T. reesei* (*xyiH*), which has high homology to known fungi D-xylulokinase genes, was silenced by transformation of * T. reesei* QM9414 strain with an antisense construct to create strain S6-2-2. The expression of the * xyiH* gene in the transformed strain S6-2-2 decreased at the mRNA level, and D-xylulokinase activity decreased after 48 h of incubation. This led to an increase in xylitol production from undetectable levels in wild-type * T. reesei* QM9414 to 8.6 mM in S6-2-2. The * T. reesei* Δxdh is a xylose dehydrogenase knockout strain with increased xylitol production compared to the wild-type * T. reesei* QM9414 (22.8 mM versus undetectable). The copy number of the xylose reductase gene (*xyl*1) in * T. reesei * Δxdh strain was increased by genetic engineering to create a new strain Δ9-5-1. The Δ9-5-1 strain showed a higher * xyl*1 expression and a higher yield of xylose reductase, and xylitol production was increased from 22.8 mM to 24.8 mM. Two novel strains S6-2-2 and Δ9-5-1 are capable of producing higher yields of xylitol. * T. reesei* has great potential in the industrial production of xylitol.

## 1. Introduction

D-Xylitol is a five-carbon polyol, which can be naturally found in various fruits and vegetables. Xylitol has similar sweetness to sucrose but lower energy value than sucrose; it has been used as sugar substitute in foods, medicine, and chemical industry. Unfortunately, the natural content of D-xylitol in fruits and vegetables is very low [1]. On an industrial scale, D-xylitol is mainly produced by chemical reduction of D-xylose from biomass hydrolysates. The biosynthesis of D-xylitol using microorganisms has gained popularity due to environmental and economic considerations using the current industrial production method [2]. Many microorganisms are able to produce D-xylitol, including bacteria, fungi, and yeasts. These include four species of* Candida, Saccharomyces, Debaryomyces, Pichia, Hansenula, Torulopsis, Kloeckera, Trichosporon, Cryptococcus, Rhodotorula, Monilia, Kluyveromyces, Pachysolen, Enterobacter liquefaciens, and Corynebacterium* spp. [3].

**Figure 1:**
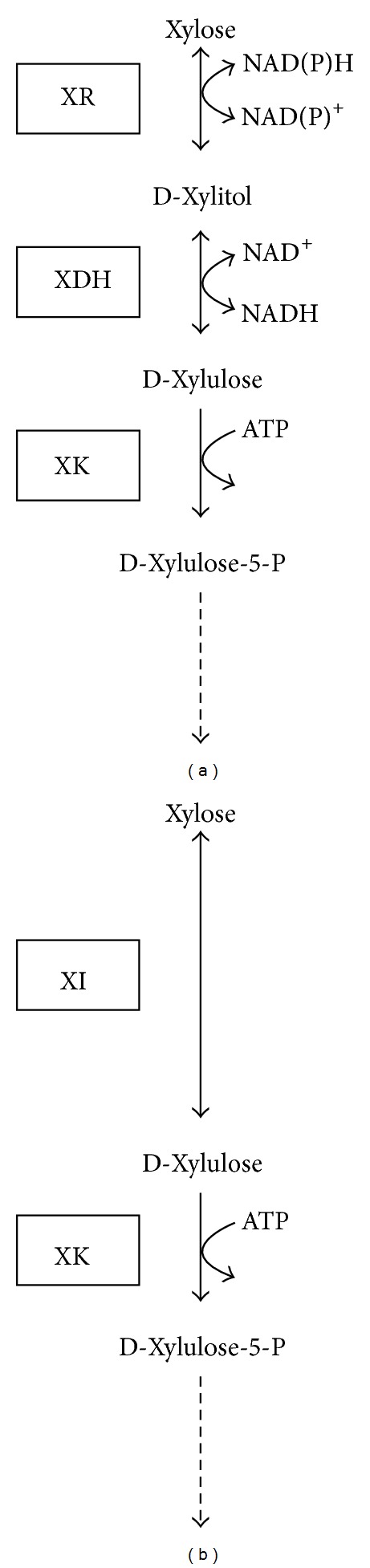
Metabolic pathway for xylose utilization. (a) The XR-XDH pathway in yeast and fungi. (b) The XI pathway in bacteria. XR: xylose reductase. XDH: xylitol dehydrogenase. XK: D-xylulokinase. XI: xylose isomerase [4].

In bacteria, D-xylose is converted to D-xylulose by xylose isomerase in a single step (Figure 1(b)). In yeasts and fungi, however, the conversion of D-xylose to D-xylulose occurs using a two-step process: a reduction step followed by an oxidation step (Figure 1(a)). D-Xylose is first reduced to D-xylitol by xylose reductase (XR) and then D-xylitol is oxidized to D-xylulose by xylitol dehydrogenase (XDH). D-Xylulose can be further metabolized to xylulose-5-phosphate by xylulokinase (XK). Xylulose-5-phosphate can enter the pentose phosphate pathway.

Lignocellulosic wastes are largely produced by a variety of industries such as forestry, agriculture, and food.* T. reesei* is an efficient biomass degrader and is a prolific industrial cellulase and hemicellulase producing fungus.

Studies have been carried out for xylitol production in yeast, especially in* Candida* species and* Saccharomyces cerevisiae* [5–7]. However, fewer studies have been completed on xylitol production in* T. reesei*. These include cloning of xylose reductase (*xyl*1) and xylitol dehydrogenase (*xdh*1) in* T. reesei *[8, 9] and antisense inhibition strategies for inhibition of xylitol dehydrogenase (XDH) in* T. reesei*. The novel strains constructed in these studies were ultimately shown to be capable of accumulating xylitol [10].

Two approaches were taken in this study in order to increase the xylitol production in* T. reesei*. In the first approach, the D-xylulokinase gene (*xyiH*) in the xylose utilization pathway in* T. reesei* was silenced; in the second one, the xylose reductase gene (*xyl*1) was overexpressed in a* T. reesei* Δxdh strain.

## 2. Materials and Methods

### 2.1. Medium

The fungal strains were grown and maintained on potato dextrose agar (PDA) containing 15.0 gL^−1^ starch, 20.0 gL^−1^ D-glucose, and 18.0 gL^−1^ agar. Strains were grown in 250 mL flasks, on a rotary shaker (200 rpm) at 30°C and in 50 mL of minimum medium containing 1.4 gL^−1^ (NH_4_)_2_SO_4_, 5 gL^−1^ KH_2_PO_4_, 0.3 gL^−1^ urea, 0.3 gL^−1^ MgSO_4_ *·*7H_2_O, 0.3 gL^−1^ CaCl_2_, 0.005 gL^−1^ FeSO_4_ *·*7H_2_O, 0.0016 gL^−1^ MnSO_4_ *·*H_2_O, 0.0014 gL^−1^ ZnSO_4_ *·*7H_2_O, and 0.002 gL^−1^ CoCl_2_, pH 5.5, with the respective carbon source.

### 2.2. Construction of Vectors

*T. reesei* genomic DNA was extracted using a Fungi/Yeast Genomic DNA Isolation Kit (Norgen Biotek, Canada). The 1932 bp D-xylulokinase gene (*xyiH*) was amplified with PCR using xyiH primers (xyiH up and xyiH down, Table S3; see Supplementary Material available online at http://dx.doi.org/10.1155/2014/169705) designed according to DNA sequence of* T. reesei* QM6a.

Fungal total RNA was extracted using an Ambion RNA extraction kit (Invitrogen, Canada) and cDNA was constructed using a Fermentas first strand cDNA synthesis kit (Fermentas, Canada). For RNA interference of the D-xylulokinase gene in* T. reesei* QM9414, plasmid pSilent-1 (Table S2) and primers S1 and S2 were used to clone the partial D-xylulokinase gene of* T. reesei* QM9414 named fragment 1. Additionally, primers S3 and S4 were used to clone the partial D-xylulokinase gene of* T. reesei *QM9414 which was named fragment 2. Fragment 1 was digested by* Xho* I and* Hind* III, inserted into pSilent-1, which resulted in pSilent-1-fragment1. Fragment 2 was digested by* Kpn* I and* Bgl* II and inserted into pSilent-1-fragment1, resulting in pSilent-xyiH.

Plasmid pPtef1-hph (Table S2) was used for* T. reesei* QM9414 xylose reductase gene overexpression. First, the hygromycin B phosphotransferase (*hph*) gene in pPtef1-hph vector was replaced by phleomycin gene (*ble*). The* ble* gene was cloned from plasmid pBC-phleo by primers ble up and ble down. Then, the* ble* gene fragment was digested by* Xba* I and* Nsi* I and inserted into plasmid pPtef1-hph, which resulted in plasmid pPtef1-ble. By using primers xyl up and A2, the xylose reductase gene (*xyl*1) was amplified. The* xyl*1 terminator was amplified using primers B1 and xyl down. Finally, these two fragments were fused using Fusion PCR. The fused fragment was digested by* Cla* I and* Hind* III and inserted into pPtef1-ble, resulting in plasmid pPtef1-ble-xyl.

### 2.3. Measurement of Xylitol

During the incubation period, aliquots of 500 *μ*L were extracted at 24 h intervals and centrifuged (16060 rcf for 5 min). The resultant supernatants were kept at 4°C for further substrate and product analyses. These supernatants were used to determine the extracellular concentration of xylitol produced by the* T. reesei* strains. The supernatant was appropriately diluted, followed by filtration using 0.2 *μ*m syringe filters (Ultident, Canada). Ultimately, they were analyzed by high-performance anion exchange chromatography with pulsed amperometric detection (HPAE-PAD) using a Dionex ICS3000 system (Dionex, Sunnyvale, USA) equipped with a 3 × 150 mm CarboPac PA20 carbohydrate column and guard. 52 mM NaOH (isocratic) eluent was used at a flow rate of 0.5 mL min^−1^ and with a full loop injection volume of 25 *μ*L. The column was maintained at 30°C, and a gold (Au) electrode with quadruple potential was used.

### 2.4. Preparation of* T. reesei* Cell-Free Extract

Mycelia were harvested by filtration and were washed extensively with cold tap water. 1 g of mycelia was blotted dry with paper towels and ground in 3 mL of extraction buffer (0.1 M Tris-HCl pH: 6.5–7.5, 1 mM EDTA, and 5 mM *β*-mercaptoethanol) to a fine powder under liquid nitrogen with mortar and pestle. Finally, the suspension was homogenized by sonication for 10 min (1 s burst and 1 s cooling period) on ice followed by centrifugation at 10,000 g for 20 min at 4°C. The supernatant was used as the cell-free extract [11].

### 2.5. Measurement of Xylose Reductase Activities

The xylose reductase activities of* T. reesei* Δxdh and Δ9-5-1 were measured using a 200 *μ*L reaction. The 200 *μ*L reaction contained 20 *μ*L cell-free extract, 50 mM sodium phosphate buffer (pH: 6.5), 100 mM D-xylose, and 0.2 mM NADPH. Background activities (without xylose) were also measured at room temperature. Absorbance changes at 340 nm were continuously monitored. One unit of xylose reductase is defined as the amount of enzyme which converts 1 *μ*M NADPH to NADP per minute at room temperature, *ε* = 6.22 mM^−1^ cm^−1^ [12]. The xylose reductase activities were normalized by the concentrations of protein.

### 2.6. Measurement of D-Xylulokinase Activities

The D-xylulokinase activities of* T. reesei* QM9414 and S6-2-2 were measured using a 200 *μ*L reaction. The 200 *μ*L reaction contained 20 *μ*L cell-free extract, 50 mM glycylglycine (pH: 7.8), 2 mM ATP, 0.5 mM PEP, 3 mM reduced glutathione, 0.1 mM NADH, 2 mM MgCl_2_, 1 mM D-xylulose, 5 mM NaF, 5 mM KCN, 10 UmL^−1^ lactate dehydrogenase, and 10 UmL^−1^ pyruvate kinase. The reaction was initiated by addition of the cell extract. Background activities (without lactate dehydrogenase and pyruvate kinase) were also measured at room temperature. Absorbance changes at 340 nm were continuously monitored. One unit of D-xylulokinase is defined as the amount of enzyme which converts 1 *μ*M NADH to NAD per minute at room temperature, *ε* = 6.22 mM^−1^ cm^−1^ [13]. The D-xylulokinase activities were normalized by the concentrations of protein.

### 2.7. Preparation of* T. reesei* Protoplast and Transformation

Protoplasts of different* T. reesei* strains used in this study were prepared according to Szewczyk et al. [14]. The protoplasts were then transformed with a pSilent-xyiH construct containing a hygromycin B phosphotransferase (*hph*) expression cassette as the selection marker or a pPtef1-ble-xyl vector containing a phleomycin (*ble*) expression cassette as the selection marker according to the method described by Szewczyk. The pSilent-xyiH transformants were screened on PDA plate containing 50 *μ*gml^−1^ hygromycin as the selection marker. The pPtef1-ble-xyl transformants were screened on PDA plate containing 100 *μ*gml^−1^ phleomycin as the selection marker. Single spore separation was done to ensure a pure culture. The integration of pSilent-xyiH into the genome of* T. reesei* QM9414 was confirmed using primers S1 and S2 (Table S3). Also, the integration of pPtef1-ble-xyl into the genome of* T. reesei* Δxdh was confirmed using ble primers (up and down) (Table S3).

### 2.8. Quantitative Real-Time PCR (qRT-PCR)

Prior to RNA extraction, mycelia were disrupted and homogenized using a motorized homogenizer (Silentcrusher M, Heidolph, Elk Grove Village, IL). Fungal total RNA was extracted using an Ambion RNA extraction kit (Invitrogen, Canada) according to the manufacturer's instructions and extracted RNA was stored at −80°C until used. The quality and integrity of the total RNA were determined using an Experion Automated Electrophoresis Station and RNA HighSens Chips (Bio-Rad, Hercules, CA). cDNA was constructed using a Fermentas first strand cDNA synthesis kit (Fermentas, Canada) according to the manufacturer's instructions and stored in −20°C until the real-time PCR analyses. The* xyiH* and* xyl*1 (target genes) gene specific primers (Table S3) were used for qRT-PCR analyses and glyceraldehyde-3-phosphate dehydrogenase (*gpd1*) primers (Table S3) were used as the housekeeping gene. qRT-PCR data were normalized using* gpd1* as the housekeeping gene. The experiments were performed using a Bio-Rad CFX96 Touch Real-Time PCR Detection System (Bio-Rad, Hercules, CA) with each well containing the following: 10 *μ*L Sso FastTM EvaGreenW Supermix (Bio-Rad, Canada), 5.0 *μ*L of appropriately diluted cDNA, 1.0 *μ*L of each primer (final concentration of 500 nM each) (Table S1), and 3.0 *μ*L of double distilled water with a total well volume of 20 *μ*L. Preliminary experiments have been done to obtain the optimal annealing temperature of each primer, the amplification efficiencies of all primers, and the optimal concentration of cDNA. Based on the preliminary results the cDNA was diluted 100-fold. The optimal RT-PCR cycling was 120 s at 98°C followed by 40 cycles of 5 s at 98°C and 5 s at 58°C. Three technical replicates were tested for each transformant to ensure consistency and accuracy. To ensure specificity of primers, melt curves were produced for each RT-PCR experiment. All primers were shown to amplify specific sequences and showed only one melting temperature on the melting curve. Serial dilutions of cDNA and a temperature gradient were used in RT-PCR in order to determine the efficiencies of all reactions and were found to be between 90 and 110% efficient.* gpd1* primers (Table S3) were used for the reference gene and data were normalized using* gpd1* primers.

For each gene (target or housekeeping) and each biological replication, ΔCt was calculated by subtracting the Ct number of the housekeeping gene from that of the target gene [15]. The ΔΔCt values were calculated using the below equation [15]:

(1) $$\begin{array}{c} \Delta \Delta \text{Ct}=\Delta \text{Ct}\;\;\text{reference}\;-\Delta \text{Ct}\;\;\text{target.} \end{array}$$

The expression ratio was calculated according to the below equation:

(2) $$\begin{array}{c} \text{Ratio}=2^{-\Delta \Delta \text{Ct}}. \end{array}$$

## 3. Results 

### 3.1. Cloning of the D-Xylulokinase Gene (*xyiH*) of* T. reesei *QM9414

**Figure 2:**
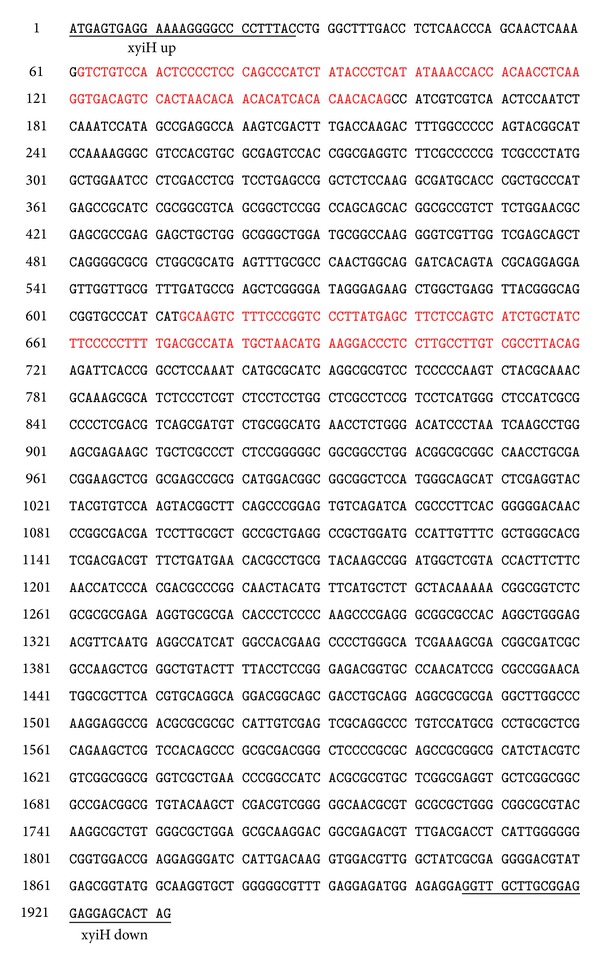
The proposed D-xylulokinase gene of* T. reesei* QM6a. Exons are shown in black while introns are presented in red color.

The genome of wild-type* T. reesei* QM6a with approximately 34 MB was subjected to genome sequence by the US Department of Energy Joint Genome Institute [16] and is available online (http://genome.jgi-psf.org/Trire2/Trire2.home.html). Because the D-xylulokinase gene sequences of fungi are known to have high similarities, the D-xylulokinase gene sequence of* Aspergillus* was used to identify a homologous gene in* T. reesei* QM6a with high similarity (about 61% of identity) to* Aspergillus* D-xylulokinase. The length of the identified gene in* T. reesei* QM6a was 1932 bp and includes two introns and motifs for the FGGY domains identified in other D-xylulokinase genes (Figure 2). Primers (Table S3) were designed from the predicted gene sequence for* T. reesei* QM6a used to amplify a PCR product of the same size (1932 bp) as the predicted sequence. The same primers (xyiH up and xyiH down) were also used to amplify the same gene from* T. reesei* QM9414 genomic DNA and the D-xylulokinase gene of QM9414 was named* xyiH*.

### 3.2. Higher Xylitol Production by* T. reesei *Strains S6-2-2 and Δ9-5-1

**Figure 3:**
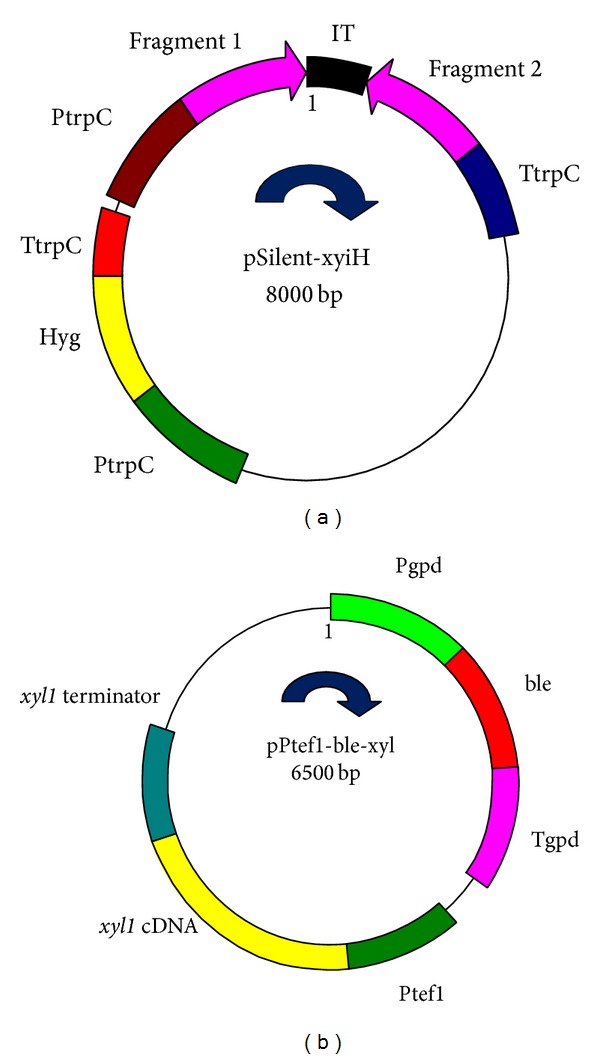
(a) The structure of pSilent-xyiH vector. PtrpC: promoter of* trpC* gene. TtrpC: terminator of* trpC* gene. Hyg: hygromycin antibiotic resistance gene. (b) The structure of pPtef1-ble-xyl vector. Pgpd: promoter of* gpd* gene. Tgpd: terminator of* gpd* gene. ble: phleomycin antibiotic resistance gene.

**Figure 4:**
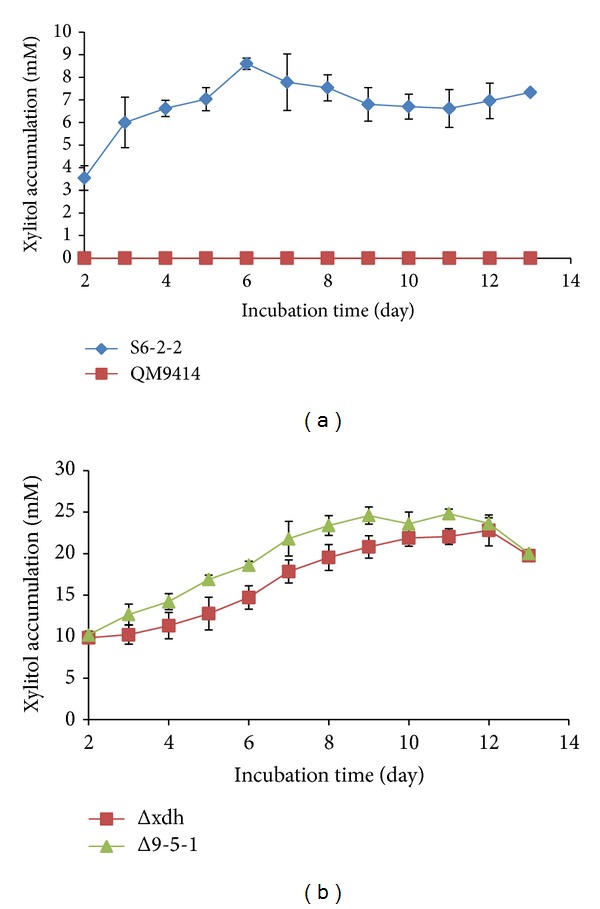
(a) Xylitol production by* T. reesei* QM9414 and S6-2-2. Red square represents* T. reesei* QM9414; green triangle represents* T. reesei xyiH* gene silenced strain S6-2-2. (b) Xylitol production by* T. reesei* Δxdh and Δ9-5-1 strains. Blue square represents* T. reesei xdh* gene knockout mutant Δxdh; purple triangle represents* xyl*1 gene overexpressed* T. reesei* Δ9-5-1 strain.

*T. reesei* QM9414 protoplast transformation with the pSilent-xyiH construct (Figure 3(a)) resulted in five clones which exhibited RNA interference of the D-xylulokinase gene. In this first approach, these five clones and the parent strain* T. reesei* QM9414 were cultured in minimum medium supplemented with xylose (25 gL^−1^) and glucose (10 gL^−1^) as carbon sources. The results showed that the highest xylitol production was achieved by one of the five positive clones named S6-2-2 (data not shown). While* T. reesei* QM9414 has no detectable xylitol production, the S6-2-2 mutant strain showed xylitol production of 8.6 mM after 6 days of incubation (Figure 4(a)). However, prolonging the incubation time (days 6 to 13) did not increase the xylitol production by* T. reesei* S6-2-2.

For the second approach to increasing xylitol production by* T. reesei*, the* T. reesei* Δxdh strain was subjected to overexpression of the xylose reductase gene. The* T. reesei* xylose dehydrogenase knockout strain Δxdh produced 22.8 mM of xylitol after 12 days of incubation. Four positive clones with enhanced copies of xylose reductase gene from the* T. reesei* Δxdh strain were obtained by transferring the pPtef1-ble-xyl cassette (Figure 3(b)) into the* T. reesei* Δxdh protoplast. The four positive clones and Δxdh were cultured in minimum medium supplemented with xylose (25 gL^−1^) and glucose (10 gL^−1^) as the sole carbon sources. The highest xylitol production was obtained for one of the four positive clones which was named Δ9-5-1, with production of 24.8 mM after 11 days of incubation (Figure 4(b)). Our results indicated that xylitol production by our transformed strains showed a similar trend as the parent strain (Figure 4(b)).

### 3.3. D-Xylulokinase Activity Measurement of* T. reesei* S6-2-2 and QM9414 Strains

**Figure 5:**
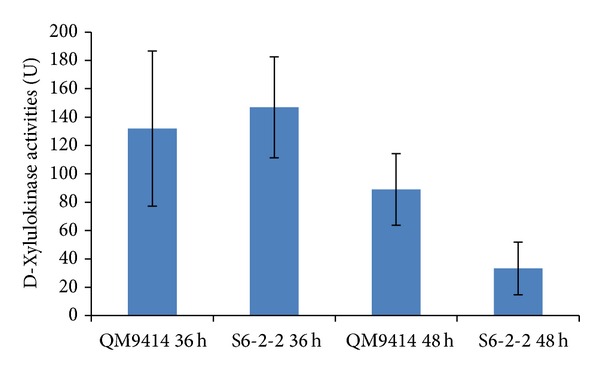
D-Xylulokinase activities of* T. reesei* QM9414 and S6-2-2 strains. D-Xylulokinase activities of* T. reesei* QM9414 and* T. reesei xyiH* gene silenced strain S6-2-2 were measured after 36 h and 48 h of incubation.

D-Xylulokinase activity was measured in* T. reesei* strains S6-2-2 and its parent strain (*T. reesei* QM9414) (Figure 5). No significant difference (*P* > 0.05) in the D-xylulokinase activities of* T. reesei* S6-2-2 and QM9414 was detected after 36 h of incubation using xylose (25 gL^−1^) and glucose (10 gL^−1^) as the sole carbon sources. However, lower D-xylulokinase activity was obtained for* T. reesei* S6-2-2 strain compared to* T. reesei* QM9414 after 48 h of incubation (*P* < 0.05) (Figure 5).

### 3.4. Xylose Reductase Activity Measurement of* T. reesei* Δ9-5-1 and Δxdh Strains

**Figure 6:**
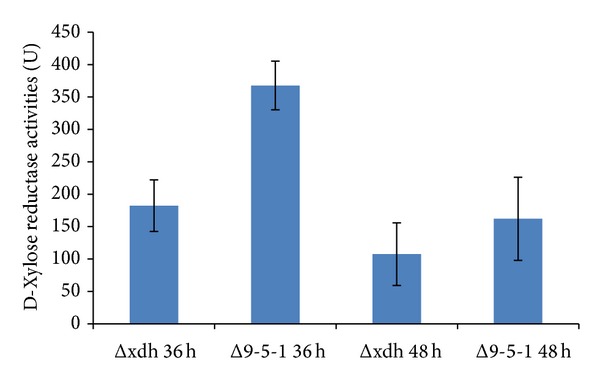
D-Xylose reductase activities of* T. reesei* Δxdh and Δ9-5-1 strains. Xylose reductase activities of* T. reesei xdh* gene knockout mutant Δxdh strain and* T. reesei xy1l* gene overexpressed Δ9-5-1 strain were measured after 36 and 48 h of incubation.

*T. reesei* strains including** Δ**9-5-1 and its parent strain Δxdh were subjected to xylose reductase activity measurement (Figure 6). Higher xylose reductase activity was obtained for the* T. reesei* Δ9-5-1 strain compared to the parent strain Δxdh using xylose (25 gL^−1^) and glucose (10 gL^−1^) as the sole carbon sources after 36 h and 48 h of incubation, especially after 36 h incubation (*P* < 0.05) (Figure 6).

### 3.5. Expression of* xyiH* and* xyl* Genes in* T. reesei *Strains S6-2-2 and Δ9-5-1

**Figure 7:**
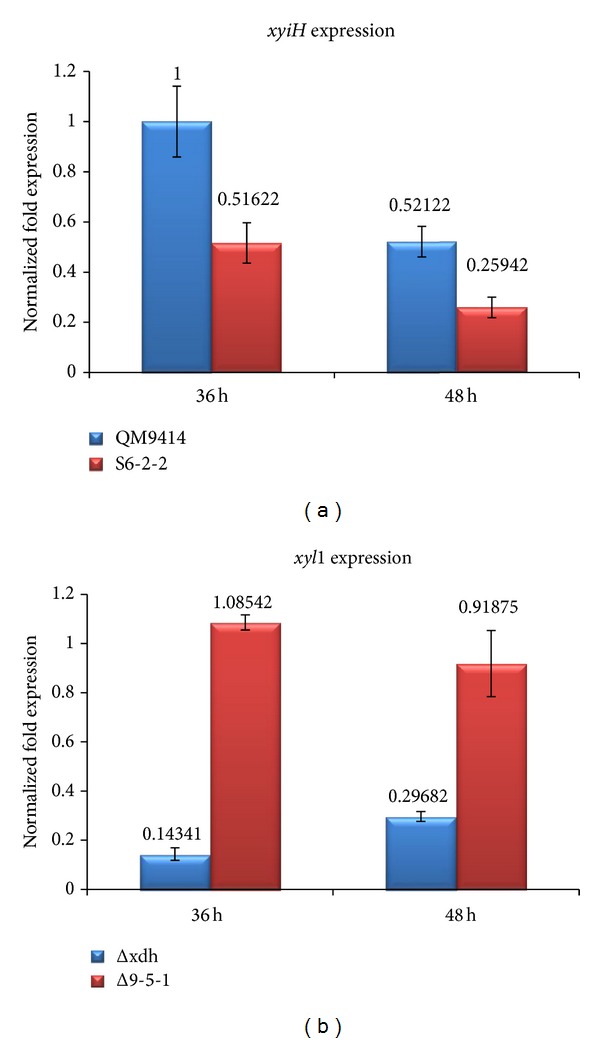
(a) D-Xylulokinase gene (*xyiH*) expression of* T. reesei* QM9414 and S6-2-2. D-Xylulokinase gene (*xyiH*) expression was measured after 36 and 48 h of incubation. (b) D-Xylose reductase gene (*xyl*1) expression of* T. reesei* Δxdh and Δ9-5-1. D-Xylose reductase gene (*xyl*1) expression was measured after 36 and 48 h of incubation.

*T. reesei* strains including S6-2-2 and Δ9-5-1 were subjected to quantitative real-time PCR (qRT-PCR) to investigate the expression levels of* xyiH* and* xyl* genes, respectively. qRT-PCR results showed that the expression of the* xyiH* gene in S6-2-2 is much lower than QM9414 after 36 and 48 h of incubation time (Figure 7(a)). This indicated that the* xyiH* gene in the* T. reesei* S6-2-2 strain was partially silenced compared to the* xyiH* expression in the parent strain* T. reesei* QM9414. This was also confirmed by the higher xylitol production (8.6 mM) of the S6-2-2 mutant strain when compared to* T. reesei* QM9414 with no detectable xylitol production (Figure 4(a)).

Additionally, the expression of the* xyl* gene in the* T. reesei* Δ9-5-1 strain was compared to its parent strain* T. reesei* Δxdh (Figure 7(b)). Our results indicated that the expression of the* xyl* gene in Δ9-5-1 is higher than* T. reesei* Δxdh after 36 and 48 h of incubation (Figure 7(b)). These are also well in line with the results obtained for the enzyme activities experiments of the* T. reesei* strains (D-xylose reductase activities, Figure 6). This was also confirmed by the high xylitol production of the xylose reductase gene (*xyl*1) overexpressed in the* T. reesei* Δxdh strain (Δ9-5-1 strain, Figure 4(b)).

### 3.6. Growth Experiments of* T. reesei* Strains

**Figure 8:**
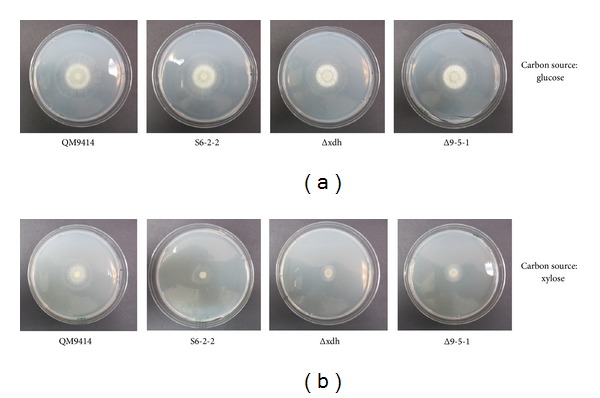
Growth of* T. reesei* QM9414, S6-2-2, Δxdh, and Δ9-5-1 using glucose (a) or xylose (b) as the sole carbon sources, respectively. S6-2-2:* T. reesei xyiH* gene silenced strain; Δxdh:* T. reesei xdh* gene knockout mutant strain; Δ9-5-1:* xyl* gene overexpressed* T. reesei* Δxdh strain.

*T. reesei* strains were grown in minimum medium containing xylose or glucose (10 gL^−1^) as the sole carbon sources. Our results indicated that* T. reesei* QM9414, S6-2-2, Δxdh, and Δ9-5-1 showed very similar growth patterns when glucose was used as the sole carbon source (Figure 8(a)). However, when xylose was used as the sole carbon source,* T. reesei* QM9414 grew faster than the other strains including S6-2-2, Δxdh, and Δ9-5-1 (Figure 8(b)). This could be a result of blocking of the xylose pathway in* T. reesei* S6-2-2, Δxdh, and Δ9-5-1 strains.

## 4. Discussion

The* T. reesei* transformants were subcultured using the antibiotic for three generations. After the third generation, genomic DNA was extracted and screened for the presence of the transformed genes using PCR. Finally, the obtained positive transformants were subjected to single spore isolation. The isolated single spores were subcultured and rescreened using gene specific primers and PCR method. Using the method, we were able to obtain stable positive transformants. Since xylose is needed for the induction and expression of* T. reesei* genes such as D-xylulokinase (*xyiH*) and D-xylose reductase (*xyl*1), cDNA should be extracted from mycelia grown using medium supplemented with xylose for the study and cloning of these genes.

Our results showed that silencing the D-xylulokinase gene in* T. reesei* was more efficient than overexpressing the xylose reductase gene (*xyl*1) with over 8 and 1.08 times xylitol production compared to levels produced by their parent strains, respectively. When D-xylulokinase gene (*xyiH*) was silenced, xylitol production increased from 0 to 8.6 mM. However, when the D-xylose reductase gene (*xyl*1) was overexpressed, xylitol production increased only marginally from 22.8 mM to 24.8 mM. This lower level of increase in xylitol production may be due to a limitation of accessible NADPH which is required for transforming xylose into xylitol. In* Candida utilis*, for example, two enzymes involved in the pentose phosphate pathway (PPP) including glucose-6-phosphate dehydrogenase and 6-phosphogluconate dehydrogenase are responsible for the production of the required NADPH [17]. A study by Ahmad et al. [5] showed that overexpression of these two enzymes led to an increase in xylitol production in* Candida tropicalis*.

Although xylitol production by* T. reesei* is not as high as that reported in yeast, the advantage of* T. reesei* is their ability to use biomass directly. Yeastscan only use biomass hydrolysates to produce xylitol or to ferment biomass with other microorganisms to produce xylitol. In contrast, genetically engineered* T. reesei* strains can use biomass directly to produce xylitol [18]. Additionally,* T. reesei* grows rapidly and there is no need for strict control of growth conditions, ultimately lowering xylitol production costs compared to the use of yeast strains. Additional improvements to improve xylitol production by* T. reesei* can be made,including genetic engineering of several genes in xylose metabolic pathway simultaneously; adjustment of fermentation conditions like carbon sources, nitrogen sources, and trace elements; and employing biomass directly to produce xylitol.

## 5. Conclusion

By using RNA interference of D-xylulokinase gene or overexpression of xylose reductase gene based on xylitol dehydrogenase knockout strain, two* T. reesei* strains S6-2-2 and Δ9-5-1 were obtained in this study. The genetically modified strains obtained have potential applications for industrial xylitol production.

## Supplementary Material

Supplementary data used in this study including strains, plasmids and primers are presented in Tables S1-3, respectively. Figure 3S represents the selection of the positive transformants obtained in this study.
