# Supplementary material for: Overexpression of D-Xylose Reductase (xyl1) Gene and Antisense Inhibition of D-Xylulokinase (xyiH) Gene Increase Xylitol Production in Trichoderma reesei
Source: Biomed Res Int. 2014 Jun 11;2014:169705. doi: 10.1155/2014/169705 (PMC4071787; doi:10.1155/2014/169705)
Supplement: Supplementary file 1 — Supplementary data used in this study including strains, plasmids and primers are presented in Tables S1-3, respectively. Figure 3S represents the selection of the positive transformants obtained in this study. [file 169705.f1.docx]

**1. 7. Supplementary data**

**Table S1.** Strains used in this study

| ***T. reesei* strains** | **Characteristics** | **Source** |
| --- | --- | --- |
| *T. reesei* QM6a | Wild-type | Provided by Dr. Irina Druzhinina, Vienna University of Technology Vienna, Austria |
| *T. reesei* QM9414 | *T. reesei* QM6a mutant with hyperactive cellulases | Provided by Dr. Tianhong Wang, Shandong University |
| *T. reesei* △xdh | *T. reesei* *xdh* knockout mutant | Provided by Dr. Bernhard Seiboth, Vienna University of Technology Vienna, Austria |
| *T. reesei* ∆9-5-1 | *T. reesei* △xdh recombinant overexpressing *xyl* gene | This work |
| *T. reesei* S6-2-2 | *T. reesei* QM9414 mutant with *xyiH* gene silenced | This work |

**Table S2.** Plasmids used in this study

| **Plasmid** | **Characteristics** | **Source** |
| --- | --- | --- |
| pPtef1-hph | Expression vector with hygromycin B resistance expression cassette (*hph*) under *gpd* promoter and terminator | Provided by Dr. Bernhard Seiboth, Vienna University of Technology Vienna, Austria |
| pBC-phleo | Expression vector containing phleomycin gene *ble* | Provided by Fungal Genetics Stock Center, USA |
| pPtef1-ble-xyl | pPtef1-hph derivative containing *xyl* gene under the control of *tef1*promoter | This work |
| pSilent-1 | Silencing vector containing hygromycin B under *trpC* promoter and terminator | Provided by Fungal Genetics Stock Center, USA |
| pSilent-xyiH | pSilent-1 derivative containing partical *xyiH* gene under the control of *trpC* promoter | This work |

**Table S3.** Primers used in this study

| **Primer** | **Sequence (5’-3’)** |
| --- | --- |
| xyl up | TCATCGATATGGCGTCTCCCACGCT |
| A2 | TCCCTTTAGCCAAACAGGTAGAGCT |
| ble up | ATTCTAGAATGGCCAAGTTGACCAGT |
| ble down | ATATGCATTCAGTCCTGCTCCTCGG |
| B1 | GCTAAAGGGACATGTTTGAGAAACAAAGAAAG |
| xyl down | TCCAAGCTTGGTCAAGCAGATTGATGC |
| xyiH up | ATGAGTGAGGAAAAGGGGCCCCTTTAC |
| xyiH down | CTAGTGCTCCTCCTCCGCAAGCAACC |
| S1 | CATCTCGAGATGAGTGAGGAAAAGGGGCCCC |
| S2 | CCCAAGCTTCCGTAACCTCAGCCAGCTTC |
| S3 | TTGGTACCATGAGTGAGGAAAAGGGGCCCC |
| S4 | GAAGATCTCCGTAACCTCAGCCAGCTTC |
| xyiH-FWD  xyiH-REV  xyl1-FWD  xyl1-REV  gpd1-FWD  gpd1-REV | CTTTACCTGGGCTTTGAC  CTTTTGGATGCCGTACTG  GCGCAGAACCTGGACAAC  AGTTTGTAGGCTTGTTGAACCG  GGAGCTCTTTGAAGAGGA  GGCAGGTACTTGACGTTTTC |

**Figure S1.** PCR selection of △xdh (pPtef1-ble-xyl) and QM9414 (pSilent-xyiH) positive transformants

Lane 1: 1kb DNA ladder

Lane 5, 6: PCR positive control: PCR amplification of *ble* gene fragment from pPtef1-ble-xyl plasmid

Lane 8: PCR amplification of *ble* gene fragment from positive transformant

Lane 9: PCR negative control: PCR amplification of *ble* gene fragment from QM9414 genomic DNA

Lane 10: PCR positive control: PCR amplification of *xyiH* gene fragment from pSilent-xyiH plasmid

Lane 11, 12, 13, 14, 15: PCR amplification of *xyiH* gene fragment from five positive transformants

Lane 16: PCR negative control: PCR amplification of *xyiH* gene fragment from QM9414 genomic DNA

**
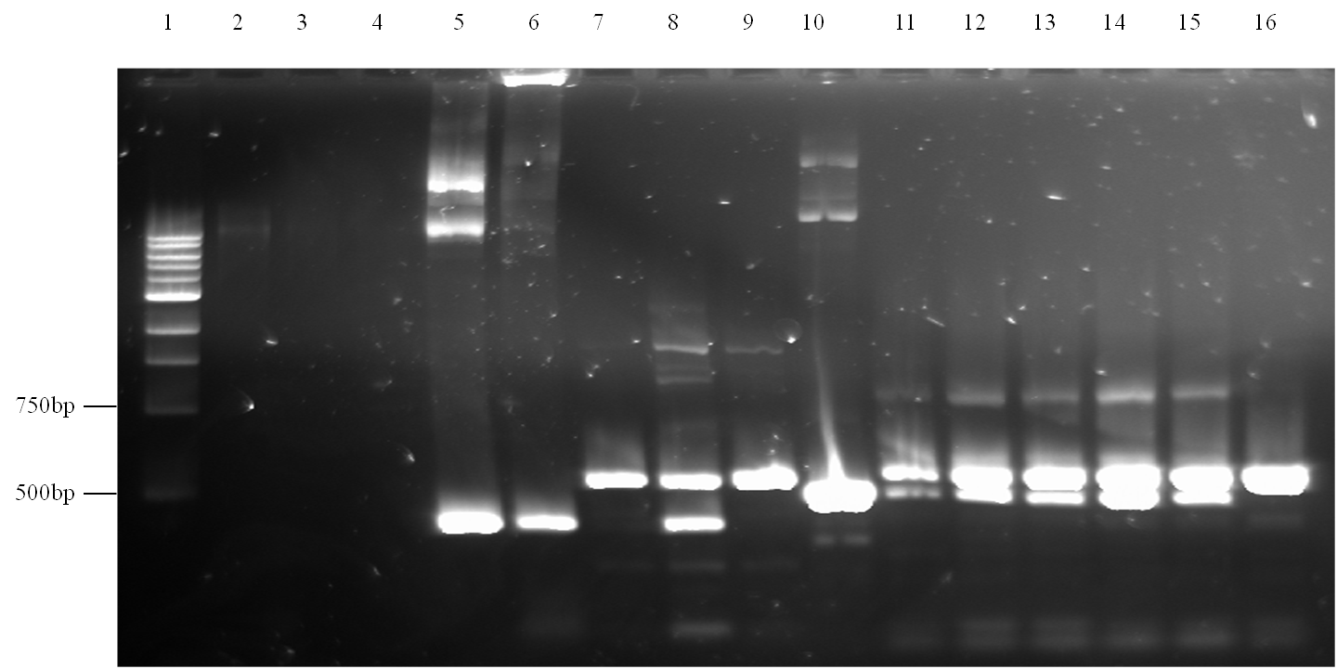
**
